# A Clustering Method of Case-Involved News by Combining Topic Network and Multi-Head Attention Mechanism

**DOI:** 10.3390/s21227501

**Published:** 2021-11-11

**Authors:** Cunli Mao, Haoyuan Liang, Zhengtao Yu, Yuxin Huang, Junjun Guo

**Affiliations:** 1 Faculty of Information Engineering and Automation, Kunming University of Science and Technology, Kunming 650500, China; maocunli@163.com (C.M.); lianghaoyuan2749@foxmail.com (H.L.); huangyuxin2004@163.com (Y.H.); guojjgb@163.com (J.G.); 2 Yunnan Key Laboratory of Artificial Intelligence, Kunming University of Science and Technology, Kunming 650500, China

**Keywords:** topic clustering, graph convolution network, multi-head attention mechanism, global feature, local feature, case-involved news

## Abstract

Finding the news of same case from the large numbers of case-involved news is an important basis for public opinion analysis. Existing text clustering methods usually based on topic models which only use topic and case infomation as the global features of documents, so distinguishing between different cases with similar types remains a challenge. The contents of documents contain rich local features. Taking into account the internal features of news, the information of cases and the contributions provided by different topics, we propose a clustering method of case-involved news, which combines topic network and multi-head attention mechanism. Using case information and topic information to construct a topic network, then extracting the global features by graph convolution network, thus realizing the combination of case information and topic information. At the same time, the local features are extracted by multi-head attention mechanism. Finally, the fusion of global features and local features is realized by variational auto-encoder, and the learned latent representations are used for clustering. The experiments show that the proposed method significantly outperforms the state-of-the-art unsupervised clustering methods.

## 1. Introduction

Public opinion analysis is an important task, the basis of which is to find out the news of the same case from a large number of case-involved news. As the case-involved news have the characteristics of sudden outbreak and rapid spread, it is hard to find out the news reports of the same cases by labeling large-scale data and training classification models [1]. The clustering task aims to find the samples with similar features [2], so we use the idea of clustering to gather the news of the same case together according to the features extracted from the news.

**Figure 1:**
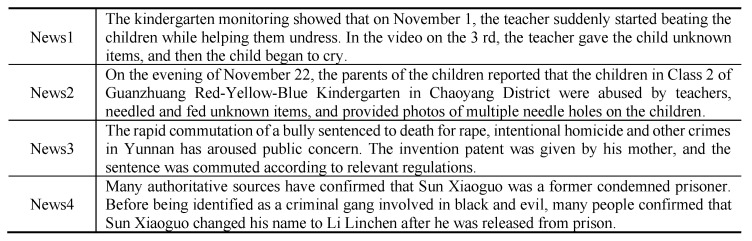
Examples of Case-Involved News.

Traditional clustering algorithms such as K-means algorithm [3] have achieved great results, but when acting on text data with sparse features, these methods are easy to converge in advance [4], resulting in unsatisfactory clustering results. Topic models can usually represent document as multinomial distribution of topics, so some researches directly apply topic models to text clustering tasks and achieve great results [5]. However, for case-involved news, there are many similar but different cases due to the proximity of topics and types. As shown in Figure 1, News1 reported the *Child Abuse Case at Xiecheng Parent-Child Garden* and News2 was the relevant report of the *Child Abuse Case at Red-Yellow-Blue Kindergarten*, the locations, personnel and event descriptions involved in the two cases were very similar. The topic models only use global information, so the extracted topic information is very similar and it is difficult to achieve its original clustering effects. In addition, with the development of neural network, deep clustering methods show superior performance. The basic idea of deep clustering is to use the generation models [6,7] such as auto-encoder(AE) to find the nonlinear mapping from raw data to latent space, then use the generated low-dimensional representations to perform clustering. However, the existing deep clustering methods only consider the local features of documents when applied to text clustering tasks.

The global features of the case-involved news refer to the relationship between the news. Based on the topic model, the topic network is constructed by using the news themselves and case information. Through the topic information and the case information, the relationships between the case-involved news in the corpus are modeled, and then the global features can be extracted by using the graph convolution network(GCN) [8]. Local features are the information carried by news text, i.e., the contextual information of the news themselves. For the local features, the multi-head attention mechanism [9] can catch the long-term dependency relationship between texts, and its powerful features extraction capability has been proved [10]. Furthermore, how to effectively combine the extracted global features and local features is crucial to the clustering task of case-involved news. As shown in Figure 1, “beating the children” appears in news 1 and “were abused by teachers” appears in news 2, and the key locations and people in the above news include “kindergarten, teacher and children”, these features are very similar after being encoded by the model. Therefore, the two news may be considered to belong to the same case, but in fact they belong to different cases, and we call them local features. In news 3 and news 4, only considering the local features, the model may not find any similarities between the two news, but human beings can judge that they describe the same case by reading a large number of such news reports. Therefore, we integrate global features and local features through variational auto-encoder, and realize the information complementation between the two features, thus solving the problem that the existing text clustering methods are hard to distinguish different cases in the same category.

Overall, the main contributions of this paper include: (1) We propose a deep clustering method of cases-involved news, which combines topic network and multi-head attention mechanism. The information complementation between global features and local features is realized through variational auto-encoder, and these features are integrated into deep clustering. (2) We propose a method to extract the global features of the cases-involved news. The topic network is constructed by integrating the case information and the contribution of different topics, thus modeling the correlation between the cases-involved news in the corpus, and then extracting the global features by using the graph convolution network.

## 2. Related Work

The clustering task of case-involved news aims to find the news sets describing the same cases. For text clustering task, existing methods can be mainly divided into topic models and deep clustering models. Topic models play an important role in multi-document contextual analysis tasks, traditional topic models (such as LDA [11]) assume that each document is a multinomial distribution of topics, and each topic is a multinomial distribution of words. With the development of pre-training model, more and more researches have begun to apply pre-training products such as word embedding to topic models to generate more coherent topics. For example, Gupta et al. [12], inspired by bidirectional language model [13] and recurrent neural network [14], proposed a topic model based on neural autoregressive distribution estimation, which effectively utilizes the context information of documents. Dieng et al. [15] introduced pre-training word embedding on the basis of LDA model, thus proposed ETM model, which effectively solved the long tail phenomenon caused by large vocabulary. Bianchi et al. [16] comprehensively considered the features of text data, fused the contextual features and statistical features of text in the model, and effectively combined the pre-training language model and the neural topic model [17]. Topic model can transform the document into multinomial probability distribution of topics, which can be regarded as the soft distribution of each cluster in clustering, so clustering can be carried out directly by topic probability. However, if we directly use the topic model to solve the clustering task of case-involved news, it will be hard to distinguish different cases under the same category because the local features of news are ignored.

Considering that the existing deep learning methods have achieved great results in local feature extraction tasks [9], we combine traditional topic clustering with deep clustering. Deep clustering models aim to treat the dimensionality reduction tasks and clustering tasks, its basic idea is that first learn reduced representations from the raw data, then utilizes the latent representations for clustering tasks. Song et al. [18] proposed an auto-encode based data clustering method, which takes into account the reconstruction loss of the auto-encoder and limits the distance between the data and the corresponding clustering center in the latent space, thus defining a new objective function, which helps the model achieve great results. Based on various variants of auto-encoder, such as stacked auto-encoder (SAE) [7], variational auto-encoder (VAE) [6], a variety of deep clustering methods have achieved good results [19,20]. In addition, how to define the loss function has always been a key problem in the researches of deep clustering. Early deep clustering methods only use the reconstruction loss of auto-encoder as the optimization objective, but this is equivalent to simply using the latent representation of auto-encoder for clustering. In order to obtain more effective representation of clustering tasks, existing researches usually add clustering loss to fine-tune the model after pre-training [19,20]. Moreover, considering that the structure existing in the data plays an important role in clustering tasks, how to use the structure information contained in the data to improve the effects of clustering model has become a research hotspot. With the development of graph neural network, the structure capture capability shown by graph neural network has achieved excellent results in more and more NLP tasks. On this basis, Wang et al. [21] proposed a goal-directed deep learning approach, which jointly optimized the embedding learning and graph clustering, to the mutual benefit of both components. Bo et al. [22] combined the latent information extracted by auto-encoder with the structural information captured by GCN for clustering, and proposed a dual self-supervised mechanism to obtain better clustering effects. Chiu et al. [23] proposed a graph-based document clustering representation method, which used keywords as nodes, the local and global features as edges to construct graph, effectively combining local and global features of documents.

In summary, under the inspiration of Bianchi et al. [16] and Chiu et al. [23], we propose a clustering method of case-involved news by combining topic network and multi-head attention mechanism according to the global features and the local features of cases-involving news.

## 3. Method

**Figure 2:**
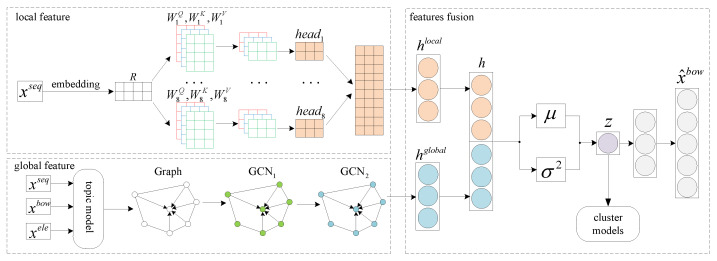
Overall framework of the proposed method.

In this section, we will introduce our proposed clustering model of case-involved news. The overall framework is shown in Figure 2, the model consists of global features extraction layer, local features extraction layer and features fusion layer. Formally, we process each news **x** into $x^{seq}$, $x^{bow}$ and $x^{ele}$. $x^{seq}\in R^{L}$ is the word index sequence vector of size *L*, where $x_{i}\in \left\{1,\ldots ,C\right\}$ is the index of the *i*-th word in the dictionary of vocabulary size *C*. $x^{bow}\in R^{C}$ is the bag-of-word(BoW) term vector of **x**. $x^{ele}=\left\{e_{1},e_{2},...,e_{n}\right\}$ is the set of case elements, where *n* is the number of case elements contained in **x**.

### 3.1. Local Feature Extraction

**Figure 3:**
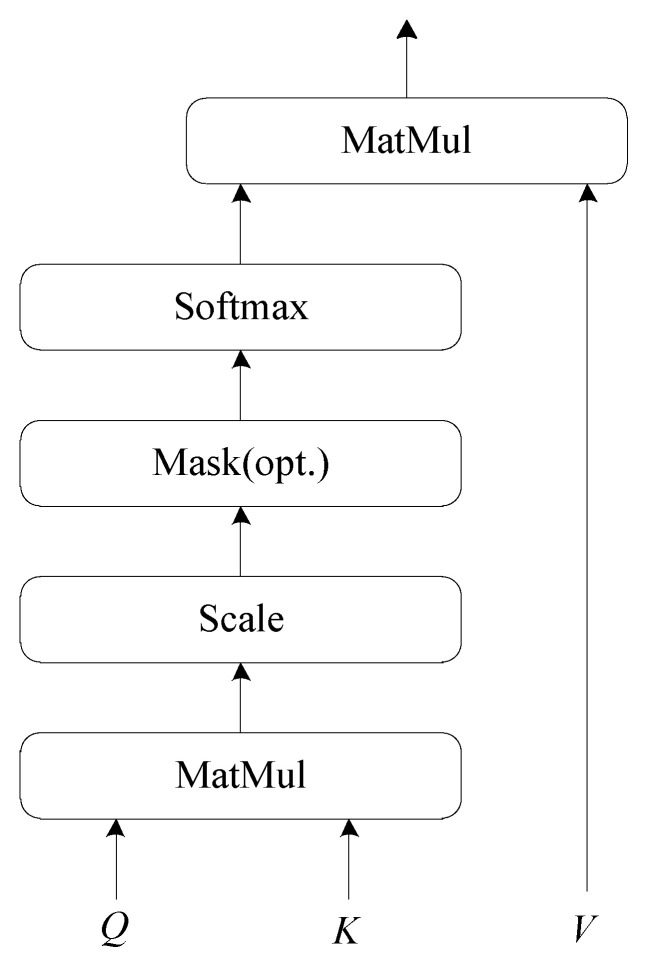
Overall architecture of self-attention mechanism.

As shown in Figure 3, the self-attention mechanism calculates the attention weights inside the text and distributes the larger weight to the more important part to find the internal relation of the text. The model converts word index sequence $x^{seq}$ into word embedding representation $R\in R^{L\times d}$, where *d* is the embedding dimension. Then, the *Q*(Query), *K*(Key) and *V*(Value) vectors are obtained through the weight matrices $W^{Q}$, $W^{K}$ and $W^{V}$:

(1) $$\left\{\begin{array}{c} Q=R*W^{Q}\\ K=R*W^{K}\\ V=R*W^{V} \end{array}\right.$$

The attention weights which represent the degree of influence of the current position word on each word in the text can be calculated as follows:

(2) $$Attention(Q,K,V)=softmax(\frac{QK^{T}}{\sqrt{d_{k}}})V$$

where $d_{k}$ is the dimension of *K*, and $\sqrt{d_{k}}$ is the scaling factor which can avoid the result of dot-product of *Q* and $K^{T}$ being too large.

Self-attention mechanism can capture the dependency relationship of words regardless of the distance between words, but a single self-attention mechanism cannot capture text features from multiple aspects. The multi-head attention mechanism can better solve this problem, the main idea is to connect the outputs of multiple self-attention mechanisms and obtain the contextual information of the text, it can be calculated as follows:

(3) $$head{}_{i}=Attention{}_{i}\left(Q_{i},K_{i},V_{i}\right)$$

(4) $$h^{local}=concat\left(head{}_{1},...,head{}_{m}\right)$$

where $head{}_{i}$ is the result calculated by the *i*-th self-attention mechanism, *m* is the number of self-attention mechanisms, concat(·) denotes the concatenation operation. Finally, $h^{local}$ is regarded as the local feature.

### 3.2. Global Feature Extraction

In order to make use of the case information and topic information of news, we propose a global feature extraction method for case-involved news. The topic model of the case-involved news is modeled based on iDocNADEe [12] and case elements contained in the news. iDocNADEe is a generative topic model which models the joint distribution p(x) of all words in the text. This is achieved by decomposing it as a product of conditional distributions i.e., $p\left(x\right)=\sum_{i=1}^{L}p(x_{i})$ and computing each autoregressive conditional via the neural networks for i∈{1,…,L}:

(5) $$\vec{h_{i}^{o}}\left(x_{<i}\right)=g(\vec{c}+\sum_{j<i}W_{:,x_{j}}^{D}+\gamma \sum_{j<i}E_{:,x_{j}})$$

(6) $$\overset{\leftarrow }{h_{i}^{o}}\left(x_{>i}\right)=g(\overset{\leftarrow }{c}+\sum_{j>i}W_{:,x_{j}}^{D}+\gamma \sum_{j>i}E_{:,x_{j}})$$

where $x_{<i}=\left[x_{1},...,x_{i-1}\right]$ and $x_{>i}=\left[x_{i+1},...,x_{L}\right]$, g(·) is a nonlinear activation function, $W^{D}\in R^{H\times C}$ is a weight matrix, $E\in R^{H\times C}$ is the pre-trained embedding matrix, $\vec{c}\in R^{H}$ and $\overset{\leftarrow }{c}\in R^{H}$ are bias parameter vectors. *H* is the number of hidden units (topics), γ is a mixture coefficient. $W_{:,x_{i}}^{D}$ is a matrix made of the i−1 first columns of $W^{D}$.

In order to integrate the case information into the hidden states of the news, we extract the case elements and calculates the hidden states:

(7) $$\vec{h_{i}^{e}}\left(e_{<i}\right)=g(\vec{c}+\sum_{j<i}W_{:,e_{j}}^{D}+\gamma \sum_{j<i}E_{:,e_{j}})$$

(8) $$\overset{\leftarrow }{h_{i}^{e}}\left(e_{>i}\right)=g(\overset{\leftarrow }{c}+\sum_{j>i}W_{:,e_{j}}^{D}+\gamma \sum_{j>i}E_{:,e_{j}})$$

where $e_{<i}\in x_{<i}$ and $e_{>i}\in x_{>i}$. We can calculate the bi-directional attention vectors of case elements as follows:

(9) $$\vec{y}=\tanh\left(\frac{1}{n}\sum_{i=1}^{n}\vec{h_{i}^{e}}\right)$$

(10) $$\overset{\leftarrow }{y}=\tanh\left(\frac{1}{n}\sum_{i=1}^{n}\overset{\leftarrow }{h_{i}^{e}}\right)$$

The attention vectors $\left[\vec{y},\overset{\leftarrow }{y}\right]$ encode the information contained in the case elements, using these vectors to weight the hidden states of the news and get the hidden state containing the case information. So we can calculate the bi-directional attention weights at the *i*-th word:

(11) $$\vec{a_{i}}=\frac{\exp(score\left(\vec{h_{i}^{o}}\left(x_{<i}\right),\vec{y}\right))}{\sum_{i=1}^{L}\exp(score\left(\vec{h_{i}^{o}}\left(x_{<i}\right),\vec{y}\right))}$$

(12) $$\overset{\leftarrow }{a_{i}}=\frac{\exp(score\left(\overset{\leftarrow }{h_{i}^{o}}\left(x_{>i}\right),\overset{\leftarrow }{y}\right))}{\sum_{i=1}^{L}\exp(score\left(\overset{\leftarrow }{h_{i}^{o}}\left(x_{>i}\right),\overset{\leftarrow }{y}\right))}$$

where $score\left(h_{i}^{o},y\right)={\left(h_{i}^{o}\right)}^{T}\cdot y$. The final weighted hidden states $\left[\vec{h_{i}},\overset{\leftarrow }{h_{i}}\right]$ are calculated from the hidden states $\left[\vec{h_{i}^{o}},\overset{\leftarrow }{h_{i}^{o}}\right]$ and the attention weights $\left[\vec{a_{i}},\overset{\leftarrow }{a_{i}}\right]$:

(13) $$\vec{h_{i}}(x_{<i},e_{<i})=\vec{h_{i}^{e}}(x_{<i})*\vec{a_{i}}\left(e_{<i}\right)$$

(14) $$\overset{\leftarrow }{h_{i}}(x_{>i},e_{>i})=\overset{\leftarrow }{h_{i}^{e}}(x_{>i})*\overset{\leftarrow }{a_{i}}\left(e_{>i}\right)$$

Therefore, the case information contained in the case elements is integrated in the hidden states through the attention mechanism. And each of the forward and backward autoregressive conditionals probability $p(x_{i})$ can be calculated as follows:

(15) $$p\left(x_{i}=w|x_{<i},e_{<i}\right)=\frac{\exp(\vec{b_{w}}+U_{w,:}\vec{h_{i}}\left(x_{<i},e_{<i}\right))}{\sum_{w^{\prime }}\exp(\vec{b_{w^{\prime }}}+U_{w^{\prime },:}\vec{h_{i}}\left(x_{<i},e_{<i}\right))}$$

(16) $$p\left(x_{i}=w|x_{>i},e_{>i}\right)=\frac{\exp(\overset{\leftarrow }{b_{w}}+U_{w,:}\overset{\leftarrow }{h_{i}}\left(x_{>i},e_{>i}\right))}{\sum_{w^{\prime }}\exp(\overset{\leftarrow }{b_{w^{\prime }}}+U_{w^{\prime },:}\overset{\leftarrow }{h_{i}}\left(x_{>i},e_{>i}\right))}$$

where w∈{1,…,C}, $\vec{c}\in R^{C}$ and $\overset{\leftarrow }{c}\in R^{C}$ are bias parameters in forward and backward passes respectively and $U\in R^{C\times H}$ is a weight matrix. Then the log-likelihood of each document can be calculated by using the bi-directional autoregressive conditional probability and taken as the optimization objective of the topic model:

(17) $$\log p\left(x\right)=\frac{1}{2}\sum_{i=1}^{L}\log p\left(x_{i}|x_{<i},e_{<i}\right)+\log p\left(x_{i}|x_{>i},e_{>i}\right)$$

In order to extract the global features of the case-involved news, we use the topic distribution and case elements to construct the correlation graph between the case-involved news, and extract the information of news nodes through the graph convolution network. Formally, consider a graph G=(V,E), where V(|V|=N) is the set of nodes and $E=\{\left(v_{i},v_{j}\right)\}$ is the set of edges. For a one-layer GCN, the new k-dimensional node feature matrix *L* can be computed as:

(18) $$L=\rho (\tilde{A}XW)$$

where $\tilde{A}=D^{-\frac{1}{2}}AD^{-\frac{1}{2}}$ is the normalized symmetric adjacency matrix, $A\in R^{N\times N}$ is the adjacency matrix of G, $D_{ii}=\sum_{j}A_{ij}$ is the degree matrix of *A* and *W* is a weight matrix, ρ is the activation function. $X\in R^{N\times M}$ is the feature matrix, where *M* is the dimension of the feature vectors.

**Figure 4:**
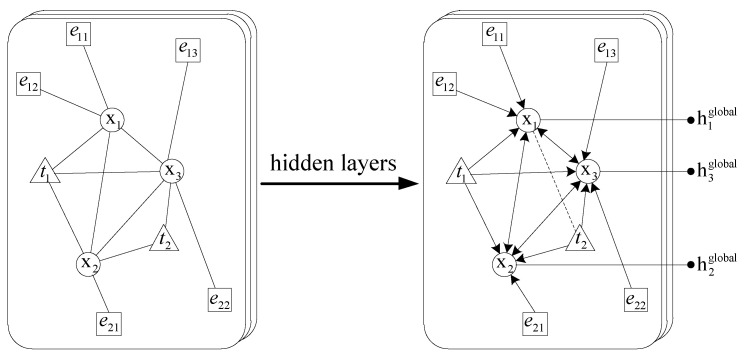
Schematic of our GCN model.

The overall our GCN model is schematically illustrated in Figure 4, where x, t, e respectively represent news set, topic set and case element set, the arrows represent the transfer of information between the nodes. Next, we will introduce in detail how to construct the graph. First, the similarity between news *i* and *j* is calculated by:

(19) $$S_{ij}={\left(x_{i}^{bow}\right)}^{T}\cdot x_{j}^{bow}$$

Then we select the top-10 similarity points of each news as its neighbors to construct an undirected K-nearest neighbor graph. Since each piece of news can be regarded as a multinomial distribution representation of topics, i.e., $x_{i}=\left[p\left(t_{1}|x_{i}\right),...,p\left(t_{H}|x_{i}\right)\right]$, we take the probability that each piece of news is assigned to each topic as the edge between news and topic nodes. Between the news nodes and the case element nodes, if a case element appears in a piece of news, an edge is built between the news node and the case element node. In this way, we can get the adjacency matrix A from the non-graph data:

(20) $$A_{ij}=\left\{\begin{array}{c} 1\;\;i,j\in x,\;j\in KNN(i)\\ p(j|i)\;i\in x,\;j\in t\\ 1\;\;i\in e,\;j\in x,\;i\in j\\ 1\;\;i=j\\ 0\;\;otherwise \end{array}\right.$$

Then the constructed graph is sent into a two-layer graph convolution network. We set the feature matrix X=I as an identity matrix which means every node is represented as a one-hot vector as the input to the model. Finally, the global features can be obtained by combining the topic information and the case information:

(21) $$h^{global}=\mathrm{ReLU}\left(\tilde{A}\mathrm{ReLU}\left(\tilde{A}XW^{0}\right)W^{1}\right)$$

where $W^{0}$ and $W^{1}$ are weight matrices of the two-layer GCN.

### 3.3. Features Fusion

In order to realize the complementarity of global features and local features, we use the variational auto-encoder to realize the fusion of the features. First of all, we concatenate the above features:

(22) $$h=concat(h^{local},h^{global})$$

Then use the final feature *h* as the input of VAE to find the latent distribution of raw data. Specifically, let *z* represents the output of the inference network, i.e., the latent representation. The VAE model assumes that the posterior probability of the input data under the latent representation approximately satisfies the Gaussian distribution, i.e., $q\left(z|h\right)∼N\left(z;\mu ,\sigma^{2}I\right)$, where $\mu ,\sigma^{2}$ are the mean and variance of the Gaussian distribution respectively. Furthermore, VAE assumes that *z* satisfies the standard Gaussian prior, i.e., p(z)∼N(0,I). In the generation phase, the VAE reconstructs the data by sampling *z*. In order to make the reconstructed data as close as possible to the raw data, VAE minimizes the error between the trained posterior probability q(z|h) and the theoretical variational probability p(z|h) while maximizing p(h):

(23) $$L_{VAE}=E_{z∼q(z|h)}\left[\log p\left(h|z\right)\right]-D_{KL}\left(q\left(z|h\right)||p\left(z\right)\right)$$

**Figure 5:**
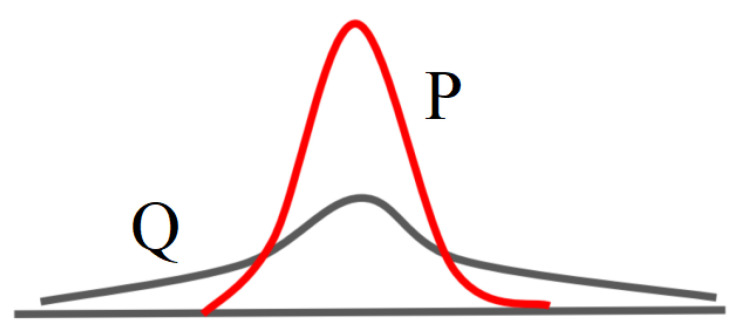
Target distribution and auxiliary distribution.

Thus, we complement the global features and local features of the case-involved news, and use the output of the inference network, i.e., *z*, as the final representation of the news, thus implementing the clustering of the case-involved news. In addition, in order to optimize the clustering effects, it is necessary to force the samples to be closer to the corresponding cluster centers, so as to realize the minimum distance within the cluster and the maximum distance between clusters. Therefore, we add clustering signals in the process of latent representation learning to obtain better clustering effects. We use a self-supervised method proposed by Xie et al. [19] to calculate the clustering loss. First use the Student’s *t*-distribution as a kernel to measure the similarity between news and centroid:

(24) $$q_{ij}=\frac{{\left(1+||z_{i}-\mu_{j}|{|}^{2}\right)}^{-1}}{\sum_{j}{\left(1+||z_{i}-\mu_{j}|{|}^{2}\right)}^{-1}}$$

where $z_{i}$ is the latent representation of $x_{i}$, $\mu_{j}$ is the center of cluster *j*, so $q_{ij}$ can be interpreted as the probability of assigning news *i* to cluster *j* (i.e., a soft assignment). Then calculate the auxiliary distribution, as shown in Figure 5, where the auxiliary distribution can be calculated as follows:

(25) $$p_{ij}=\frac{q_{ij}^{2}/\sum_{i}q_{ij}}{\sum_{j}\left(q_{ij}^{2}/\sum_{i}q_{ij}\right)}$$

where $q_{ij}^{2}$ achieves the effect of emphasis, making the distribution more credible. On this basis, clustering loss is defined as KL divergence between the two distributions:

(26) $$L_{clustring}=KL\left(P||Q\right)=\sum_{i}\sum_{j}p_{ij}\log\frac{p_{ij}}{q_{ij}}$$

In summary, the training process is divided into two steps:

- pre-training. The extracted local features and global features are concatenated as the input of the variational auto-encoder, and Equation (23) is taken as the optimization target for iterative training. After the pre-training, the latent representations of the news can be preliminarily obtained.
- fine-tuning. Firstly, we use the K-means model to initialize the cluster center on the basis of step 1, and then the clustering loss $L_{clustering}$ is calculated according to Equations (24)–(26). Finally, the loss function is defined as:
  (27) $$L=\left(1-\lambda \right)\cdot L_{VAE}+\lambda \cdot L_{clustring}$$
  where λ is the weight which balances the above two losses. The purpose of this step is to add clustering signals in the training process so as to obtain the latent representations of case-involved news that is more suitable for clustering.

## 4. Experiment

### 4.1. Dataset

When building the dataset, we follow the following standards: 1. Case-involved events in recent years; 2. Number of public opinions with a certain scale; 3. From official media. We analyzed the hot cases in recent years and selected several high-profile cases to collect data. After that, we manually analyzed the collected news and established the corresponding relationship between the case and the news, and a piece of news was only related to one case. In addition, we also applied some standardized text preprocessing steps, including tokenization [24], special character cleaning and so on. Finally, 12,468 case-involved news items were collected from Chinese microblog Weibo (https://m.weibo.cn/, accessed on 20 October 2021) and official websites of major news media, like Xinhuanet (http://xinhuanet.com/, accessed on 20 October 2021), The People’s Daily (http://paper.people.com.cn/, accessed on 20 October 2021) and so on. The final dataset including five homicide cases, four infringement cases, four fraud cases, one bombing case and one indecent case. The statistics information of our dataset is presented in Table 1.

As the embodiment of the key information of the case, there have been many researches aimed at exploring the relationship between the case elements and the case. For example, Han et al. [25] have explored the case elements in the news and believed that different news of the same case often has different emphases. Combined with the task of this article, we use this method to define and extract the case elements.

### 4.2. Baseline Models

In order to prove the effectiveness of the proposed method, the following baseline models are selected in this paper, including traditional clustering, deep clustering and clustering based on the structural of data.

- K-means [3]: A classical clustering method based on raw data;
- AE [18]: It is a two-stage deep clustering algorithm which performs K-means on the representations learned by autoencoder;
- DEC [19]: It is a deep clustering method which designs a clustering objective to guide the learning of the data representations;
- DCN [20]: This method adds the objective function of Kmeans algorithm to AE;
- IDEC [26]: This method adds a reconstruction loss to DEC, so as to learn better representation;
- N2D [27]: It is a unsupervised method which carries out manifold learning on the basis of raw data and auto-encoder;
- SDCN [22]: This method integrates the data representation obtained from the encoder and the structural information extracted from the graph convolution network, and designs a dual self-supervised clustering guidance method.

### 4.3. Experiment Details

- **Experimental environment**: In this paper, we implement the experiments in python3.6.5, pytorch1.6.0 [28] on a NVIDIA TESLA T4.
- **Corpus processing**: For the corpus, we construct a vocabulary with size of 29,651 by selecting words with a frequency greater than three and removing stop words. For each news, we add the [CLS] flag at the beginning of each news as the starting flag, and the model uses this flag to extract the local feature. Otherwise, we intercept the part with the length of more than 100, and use the [pad] flag to pad the news with the length of less than 100 which is conducive to using the mask mechanism to eliminate invalid information. The adjacency matrix is constructed by the method described in Section 3.2, which is a sparse matrix containing news nodes, case element nodes and topic nodes, the information contained in it can be extracted by GCN model.
- **Hyperparameters**: For local feature, we use the multi-head attention mechanism, and the number of head is 8 and the dimension of hidden layser is 512. For global feature, we set the number of topic number to 15, γ=1.0 and the dimension numbers of the 2-layer GCN model are 2000 and 512. For training process, we use the optimizer Adam [29] with learning rate of $3\times 10^{-3}$, and the number of dropout is set to 0.8. Some parameters, such as latent representation dimension and loss balance weight, will be compared with different values in the experimental part.
- **Baseline models**: For baseline models, K-means model is used for clustering, which is also the choice of the all original papers. For text representation, we chose to use the one-hot vector processed by L2 regularization, the reason for this choice is that we found that the effect of the original one-hot vector without processing is not as good as that after L2 regularization. Otherwise, we set the output dimension of AE to 20, and other parameters followed the settings of the original papers.
- **Training steps**: In the training phase, word sequence and adjacency matrix are used as inputs to the model, through multi-head attention mechanism and GCN model, model can extract the local and global features of the news. By concatenating the two features, the model obtains the input of the variational auto-encoder, then the VAE model will reconstruct the analog data, and the goal of reconstruction is the BoW (bag of word) representation of news. Finally, the method described in the last part of Section 3.3 is used to adjust the parameters, so as to obtain the representation with the best clustering effect.

### 4.4. Metrics

In order to evaluate the performance of the proposed model, we use three commonly used clustering metrics following previous researches: (*y* is the true group label, *c* is the clustering group label):

- **Accuracy**
  ACC measures the consistency between the true group label and the clustering group label. It is defined as follows:
  (28) $$ACC=\max_{m}\frac{\sum_{i=1}^{N}\delta (y_{i}=map\left(c_{i}\right))}{N}\;$$
  where δ(·) is an indicator function, map(·) transforms the clustering label $c_{i}$ to its group label by the Hungarian algorithm [30].
- **Normalized Mutual Information** [31]
  NMI is a popular metric used for evaluating clustering tasks. It is defined as follows:
  (29) $$NMI=\frac{I(y,c)}{\sqrt{H(y)H(c)}}$$
  where I(·) is mutual information which measures the information gain to the true partition after knowing the clustering result, H(·) is entropy and the denominator $\sqrt{H(y)H(c)}$ is used to normalize the mutual information to be in the range of [0, 1]. When we partition the news perfectly, NMI score is 1.
- **Average Rand Index** [32]
  ARI is defined as follows:
  (30) $$ARI=\frac{RI-E[RI]}{\max(RI)-E[RI]}$$
  where E[RI] is the expectation of the Rand index (RI), which can be calculated as shown in Equation (31). RI has a value between 0 and 1, with 0 indicating that the two data clusterings do not agree on any pair of points and 1 indicating that the data clusterings are exactly the same.
  (31) $$RI=\frac{a+b}{a+b+c+d}$$
  where a,b,c,d is defined as follows:
  - *a*: the number of pairs of news that are in the same cluster in *y* and in the same cluster in *c*;
  *b*: the number of pairs of news that are in the different clusters in *y* and in the different clusters in *c*;
  *c*: the number of pairs of news that are in the same cluster in *y* and in the different clusters in *c*
  *d*: the number of pairs of news that are in the different clusters in *y* and in the same cluster in *c*

## 5. Analysis of Results

### 5.1. Analysis of Clustering Results

In the first experiment, we compared the effects of the method proposed in this paper with the baseline models. Note that we set the dimensions of all latent representations to 20.

According to the results in Table 2, it can be seen that the K-means model using the raw data has the worst effects, in our opinion, K-means is a powerful model, but this does not mean that it can be applied to any data, unprocessed high-dimensional data can not play its powerful performance, which is also the reason for its worst effect. Therefore, the AE+KM model optimizes the Kmeans, uses the excellent data dimensionality reduction ability of the auto-encoder, and maps the row data to the low-dimensional space without losing too much information, this measure also greatly improves the effect of the model. In addition to using the latent representation of data with lower dimensions, another measure can improve the clustering effect, make full use of the optimization goal of clustering, like DEC and DCN. Based on the auto-encoder, Dec and DCN respectively design the optimization strategy suitable for deep clustering, Dec designs an auxiliary distribution to help the clustering model find the optimal cluster center, and DCN directly takes the cluster center as trainable parameters and optimizes them together in the training process. Therefore, they have achieved better results, which also proves that introducing clustering loss or clustering objective function into the model as a kind of supervision information and fine-tuning it can make the model obtain a latent representation more conducive to clustering. What the above two models have in common is that after pre-training the models by using the reconstruction loss of auto-encoder, they only use the designed optimization objectives to fine-tuning the model, which also leads to the possible deviation of the model from the original data distribution, so that IDEC achieves better performance than them by adjusting reconstruction loss and clustering loss. Considering that the essence of clustering task is to find similar samples from the data set according to the similarity, SDCN uses the idea of graph and cosine similarity to explicitly model the association relationship between samples. Our model draws on this idea, explores the correlation between the case-involved news, integrates the local and global features of the news, and integrates the context information, case information and topic information into deep clustering, so it obtains the best effect.

In Table 3, in order to more intuitively show the result analysis in Table 2, we show the differences between each model from the following four aspects: 1. Whether the model uses the dimension reduced data; 2. Whether the model uses clustering objectives for optimization; 3. Whether the model considers the correlation between samples; 4. Whether the model uses contextual information. It is precisely because we comprehensively consider the above four aspects that we design a clustering method by integrating local features and global features, so as to obtain the best effect.

### 5.2. Analysis of Latent Representation in Different Dimensions

In order to investigate whether our model is affected by different dimensions, we changed the dimensions of latent representation while leaving other parts unchanged.

As shown in Table 4, it can be seen that the results obtained by clustering with the latent representation of different dimensions are different. When the dimension is set to 128, the results obtained by the model are the worst. With the decrease of the dimension of latent representation, the model effects show an upward trend, and the clustering effects reach the best when the dimension is set to 32 and 20. However, when the dimension continues to decrease until 10, the model effects begin to show a downward trend. In view of the above phenomenon, we believe that this is because 128-dimensional representation contain a lot of useless information, and the model cannot use these information to cluster news, resulting in the worst effects of the model. When the dimension is reduced to 32 and 20, the information contained is beneficial to clustering, so the best effects are achieved. When the representation dimension is continuously reduced to 10, some useful information is missing from the representation, which is also not conducive to clustering. In summary, we conclude that it is effective to extract the latent representation of news by using the local features and global features of the case-involved news, but the effects of the model are affected by the dimension of the latent representation. If the dimension is too high, the information that is not conducive to clustering will be mixed together; if the dimension is too low, the information that is conducive to clustering will be lacking.

### 5.3. Analysis of Balance Weight λ

In this experiment, we will explore how our model is affected by different λ. In detail, we set λ=[0.0,0.1,0.3,0.5,0.7,0.9,1.0]. Note that λ=0.0 means that the representations in the model do not contain the information from K-means and λ=1.0 represents that the model only use the features learned by VAE.

From the results in Table 5, it can be seen that different λ lead different results. When λ<0.7, with the increase of λ, the effects of the model are getting better and better, and the effects are the best when λ=0.7, then with the continuous increase of λ, the effects of the model decrease instead. We believe that this phenomenon is mainly because λ is a balance weight which balances the reconstruction loss and the clustering loss. The purpose of reconstruction loss is to obtain high-quality latent representations, and clustering loss is to add supervision signals to the model. When λ is too low, the supervision signals generated by clustering loss is too weak to guide the model to obtain a more suitable data representations for clustering. As λ continues to improve, the model loses the reconstruction loss, so the effects decreases again. In summary, it is effective to fine-tune the model by using clustering loss, but the model cannot be trained by using clustering loss alone.

### 5.4. Analysis of Different Features

In order to prove the effectiveness of each feature extracted in our method, this experiment compares the effects of clustering each feature directly.

According to the results in Table 6, it can be seen that only using topic information for clustering has the worst effects. Although the topic model has good effects when applied to clustering tasks, as mentioned in Section 1, due to the particularity of the tasks and data in this paper, the topic model cannot distinguish different but similar cases well. Clustering using local features and global features respectively have achieved better results than topic model. Among them, the global features are the structural relationship between news, which is based on topic information and case information, so the effects are better. This also proves that the global feature extraction method of case-involved news proposed in this paper is indeed effective. In addition, using multi-features fusion for clustering has the best effects, which also proves that combining contextual information, topic information and case information of the case-involved news is indeed helpful for clustering the case-involved news.

## 6. Conclusions

In this paper, we propose a novel model of integrating topic network and mutli-head attention mechanism into deep clustering framework to deal with the task of case-involved news clustering. Experimental results demonstrate that using VAE to realize the information complementarity between local features and global features of case-involved news is indeed helpful to clustering tasks. In the subsequent work, in addition to the case elements, other domain knowledge, such as judgment documents, legal provisions, etc. can also be considered for case-involved news clustering task.

## Figures and Tables

**Table 1:** Data statistics for our Dataset. Where $N_{case}$ is the number of the cases, $N_{news}$ is the number of the news, $N_{maxCase}$ is the maximum number of news in the same case, $N_{minCase}$ is the minimum number of news in the same case, $N_{vocab}$ is the size of vocabulary, $N_{maxLen}$ is the maximum length of news and $N_{avgLen}$ is the average length.

$N_{\mathrm{case}}$	$N_{\mathrm{news}}$	$N_{\mathrm{maxCase}}$	$N_{\mathrm{minCase}}$	$N_{\mathrm{vocab}}$	$N_{\mathrm{maxLen}}$	$N_{\mathrm{avgLen}}$
15	12,468	1598	500	29,651	446	146

**Table 2:** Clustering results on different models.

*Model*	*ACC*	*NMI*	*ARI*
KM	0.6416	0.6692	0.5067
AE+KM	0.7271	0.7393	0.6239
DEC	0.7765	0.7487	0.6945
DCN	0.7994	0.7786	0.7112
IDEC	0.8186	0.7902	0.7456
N2D	0.8466	0.8209	0.7819
SDCN	0.9014	0.8684	0.8368
Ours	**0.9451**	**0.9011**	**0.9014**

**Table 3:** Comparison of features of each model.

*Model*	*Feature Compression*	*Clustering Optimization*	*Correlation*	*Context*
KM	-	-	-	-
AE+KM	✓	-	-	-
DEC	✓	✓	-	-
DCN	✓	✓	-	-
IDEC	✓	✓	-	-
N2D	✓	-	-	-
SDCN	✓	✓	✓	-
Ours	✓	✓	✓	✓

**Table 4:** Clustering results with different dimensions.

*Dimension*	*ACC*	*NMI*	*ARI*
10	0.9000	0.8940	0.8776
20	**0.9451**	0.9011	**0.9014**
32	0.9339	**0.9058**	0.8975
64	0.8880	0.8736	0.8509
128	0.8586	0.8483	0.8227

**Table 5:** Clustering results with different λ.

*λ*	*ACC*	*NMI*	*ARI*
0.0	0.9287	0.8785	0.8696
0.1	0.9261	0.8743	0.8612
0.3	0.9301	0.8796	0.8741
0.5	0.9347	0.8858	0.8821
0.7	**0.9451**	**0.9011**	**0.9014**
0.9	0.9394	0.8929	0.8913
1.0	0.9354	0.8897	0.8794

**Table 6:** Clustering results with different features.

*Feature*	*ACC*	*NMI*	*ARI*
topic information	0.7495	0.7179	0.6408
local feature	0.8587	0.8333	0.8267
global feature	0.8829	0.8658	0.8428
feature fusion	**0.9451**	**0.9011**	**0.9014**
